# Exploration and validation of a novel reactive oxygen species–related signature for predicting the prognosis and chemotherapy response of patients with bladder cancer

**DOI:** 10.3389/fimmu.2024.1493528

**Published:** 2024-12-19

**Authors:** Yulei Li, Lulu Zhang, Gang Xu, Gang Xu, Jiajun Chen, Keyuan Zhao, Mengyao Li, Jing Jin, Chao Peng, Kaifang Wang, Shouhua Pan, Ke Zhu

**Affiliations:** ^1^ Department of Urology, Shaoxing People’s Hospital, Zhejiang, Shaoxing, China; ^2^ Medical Research Center, Shaoxing People’s Hospital, Zhejiang, Shaoxing, China; ^3^ Department of Urology, Nanchang People’s Hospital, Nanchang, China; ^4^ Department of Pathology, Shaoxing People’s Hospital, Zhejiang, Shaoxing, China; ^5^ Cancer Centre, Faculty of Health Sciences, University of Macau, Macau, Macao SAR, China

**Keywords:** bladder cancer, reactive oxygen species, prognostic signature, chemotherapy response, overall survival, AKR1B1

## Abstract

**Background:**

Reactive Oxygen Species (ROS), a hallmark of cancer, is related to prognosis, tumor progression, and treatment response. Nevertheless, the correlation of ROS-based molecular signature with clinical outcome and immune cell infiltration has not been thoroughly studied in bladder cancer (BLCA). Accordingly, we aimed to thoroughly examine the role and prognostic value of ROS-related genes in BLCA.

**Methods:**

We obtained RNA sequencing and clinical data from The Cancer Genome Atlas (TCGA) for bladder cancer (BLCA) patients and identified ROS-associated genes using the GeneCards and Molecular Signatures Database (MSigDB). We then analyzed differential gene expression between BLCA and normal tissues and explored the functions of these ROS-related genes through Gene Ontology (GO), Kyoto Encyclopedia of Genes and Genomes (KEGG), and Protein-Protein Interaction (PPI) analysis. Prognostic ROS-related genes were identified using Univariate Cox regression (UCR) and LASSO analyses, which were further refined in a Multivariate Cox Regression (MCR) analysis to develop a Prognostic Signature (PS). This PS was validated in the GSE13507 cohort, assessing its predictive power with Kaplan-Meier survival and time-dependent ROC curves. To forecast BLCA outcomes, we constructed a nomogram integrating the PS with clinical variables. We also investigated the signature’s molecular characteristics through Gene Set Enrichment Analysis (GSEA), Immune Cell Infiltration (ICI), and Tumor Mutational Burden (TMB) analyses. The Genomics of Drug Sensitivity in Cancer (GDSC) database was used to predict chemotherapy responses based on the PS. Additionally, we screened for Small-Molecule Drugs (SMDs) targeting ROS-related genes using the CMAP database. Finally, we validated our findings by checking protein levels of the signature genes in the Human Protein Atlas (HPA) and confirmed the role of Aldo–keto reductase family 1 member B1 (AKR1B1) through *in vitro* experiments.

**Results:**

The constructed and validated PS that comprised 17 ROS-related genes exhibited good performance in predicting overall survival (OS), constituting an independent prognostic biomarker in BLCA patients. Additionally, we successfully established a nomogram with superior predictive capacity, as indicated by the calibration plots. The bioinformatics analysis findings showcased the implication of PS in several oncogenic pathways besides tumor ICI regulation. The PS was negatively associated with the TMB. The high-risk group patients had greater chemotherapy sensitivity in comparison to low-risk group patients. Further, 11 candidate SMDs were identified for treating BLCA. The majority of gene expression exhibited a correlation with the protein expression. In addition, the expression of most genes was consistent with protein expression. Furthermore, to test the gene reliability we constructed, AKR1B1, one of the seventeen genes identified, was used for in-depth validation. *In vitro* experiments indicate that siRNA-mediated AKR1B1 silencing impeded BLCA cell viability, migration, and proliferation.

**Conclusions:**

We identified a PS based on 17 ROS-related genes that represented independent OS prognostic factors and 11 candidate SMDs for BLCA treatment, which may contribute to the development of effective individualized therapies for BLCA.

## 1 Introduction

Bladder cancer (BLCA) has the sixth worldwide prevalence of new cases and the ninth-highest number of fatalities among male cancer patients globally. In 2020, there were nearly 573,000 new cases and nearly 213,000 deaths caused by BLCA (1, 2). Based on the depth of muscle invasion, BLCA can be mainly classified into non-muscle-invasive BLCA (NMIBC) and muscle-invasive BLCA (MIBC) (3). Despite remarkable advancements in treatments, including adjuvant chemotherapy, immune checkpoint inhibitor therapy, robot-assisted surgery systems, and targeted therapy, the overall survival (OS) of BLCA patients remains unfavorable (4–6). In addition, BLCA is a cancerous malignancy with notable and substantial heterogeneity, and conventional clinical predictive factors, including tumor grade and TNM stage, can be utilized for predicting BLCA patient prognosis accurately (7). Hence, identifying novel biomarkers for predicting the BLCA patient survival time is of crucial practical clinical significance.

Reactive oxygen species (ROS), characterized by molecules that contain oxygen with oxidizing properties, are the reduction products of oxidative metabolism and consist of nonradicals, mainly hydrogen peroxide (H2O2), hypochlorous acid (HOC1), and organoid hydroperoxides (ROOH), and free radicals, mainly hydroxyl and superoxide anion radicals (8). Mitochondria, peroxisomes, the endoplasmic reticulum (ER), metabolic enzymes, and the Warburg effect are the main endogenous sources of ROS (9). ROS can also be produced by physical agent exposure (ultraviolet rays and heat), chemotherapy, and radiotherapy (10, 11). ROS has been indicated to be crucial secondary messengers governing various cellular biological processes, including proliferation, angiogenesis, differentiation, metastasis, autophagy, drug resistance, immune response, and cancer stem cells (12). Moderate ROS levels are believed to be essential for cell growth and differentiation. Nevertheless, the excessive accumulation of ROS is involved in multiple diseases (13), particularly malignant tumors (14, 15). Recent studies have indicated that an imbalance in ROS is closely related to BLCA development and progression (16, 17). Therefore, comprehensively investigating the functions of ROS-related genes and identifying ROS-related biomarkers to accurately predict BLCA patients’ OS is highly important.

The relationship between genes and reactive oxygen species (ROS) is multifaceted, including the regulation of ROS production and clearance by genes, and the influence of ROS on gene expression (18). Here are some key points that outline the interaction between genes and ROS: (1) Regulation of ROS by Genes. Genes such as p53 play a critical role in maintaining genomic integrity and orchestrating cellular responses to stress, including the modulation of ROS activity. ROS can act as signaling molecules to initiate p53 activation in response to DNA damage, leading to transcriptional regulation of genes involved in cell cycle arrest, DNA repair, and apoptosis (19, 20). (2) ROS Influence on Gene Expression. The Keap1-Nrf2-ARE signaling pathway is a well-studied regulatory system that preserves cellular redox homeostasis (21, 22). ROS act as central players in this mechanism, providing a dynamic balance between Nrf2 activation and its inhibition by Keap1. When cellular ROS levels rise, certain cysteine residues in Keap1 are oxidized, disrupting its ability to ubiquitinate Nrf2, leading to the accumulation of Nrf2 in the nucleus and the transcriptional activation of antioxidant and detoxification genes (22). (3) ROS and Chromatin. ROS influence the activity of epigenetic modulators, such as histone deacetylases (HDACs) or DNA methyltransferases (DNMTs), affecting the expression of target genes. They also oxidize DNA, particularly adenine and guanine, which can lead to mutations and contribute to tumorigenesis (23, 24). (4) ROS and Cancer. In cancer therapy, ROS can either activate or suppress NF-kB signaling involved in the control of cellular processes such as embryogenesis, cell proliferation and death, and responses to stress stimuli (21). Additionally, ROS can induce DNA hypermethylation, potentially affecting tumor phenotype when promoter regions of tumor suppressor genes are involved (25).

Our study comprehensively investigated the functions and prognostic values of ROS-associated genes in BLCA by accessing a public database via bioinformatics methods, aiming at constructing and validating a novel Prognostic Signature (PS) relying on ROS-related genes in BLCA through LASSO and Cox regression analyses. We also explored the associations between PS and Immune Cell Infiltration (ICI), Tumor Mutational Burden (TMB), and chemosensitivity. A nomogram was established by combining the Risk Scores (RSs) based on the seventeen prognostic ROS-associated genes and clinical characteristics. Additionally, we identified 11 candidate Small-Molecule Drugs (SMDs) for BLCA treatment. To verify the authenticity of the data, *in vitro* experiments revealed that siRNA-mediated AKR1B1 silencing impeded BLCA cell viability, migration, and proliferation, aligning with our expectations and demonstrating the constructed ROS-related gene reliability. We identified a PS based on 17 ROS-related genes that represented independent OS prognostic factors and 11 candidate SMDs for BLCA treatment, which may contribute to the development of effective individualized therapies for BLCA.

## 2 Methods and methods

### 2.1 Data acquisition

We first obtained ROS-related genes from the GeneCards database (https://www.genecards.org/) and Molecular Signature Database v7.1 (MSigDB; https://www.gsea-msigdb.org/gsea/msigdb). Then, we downloaded the level-three transcriptome RNA sequencing information and clinicopathological features of BLCA patients by accessing The Cancer Genome Atlas (TCGA) (https://gdc‐portal.nci.nih.gov/). Further, we utilized the GSE13507 acquired from the Gene Expression Omnibus database (GEO, https://www.ncbi.nlm.nih.gov/geo/) as the validation set.

### 2.2 Identification of ROS‐associated differentially expressed genes

Employing the R edge package (version R 4.0.5, https://bioconductor.org/packages/release/bioc/), the ROS‐related DEGs between BLCA and normal bladder samples were screened, setting the cutoff criteria as a False Discovery Rate (FDR) < 0.05 and a |log2-fold change (FC)| > 1.

### 2.3 Enrichment analysis of ROS‐related DEGs

Gene Ontology (GO) analysis that includes molecular function (MF), cell component (CC), and biological process (BP) analyses was implemented to explore the possible molecular mechanisms behind ROS‐related DEGs via the clusterProfiler package of R, utilizing the same approach for Kyoto Encyclopedia of Genes and Genomes (KEGG) analysis (26–28) and considering *P* < 0.05 as significant enrichment.

### 2.4 Protein-protein interactions

ROS‐related DEGs were uploaded to the STRING database (http://www.string-db.org/) to obtain PPI information. The PPI network establishment and visualization were conducted via Cytoscape software, using the MCODE plug-in to screen the considerable PPI network modules.

### 2.5 Identification of potential small-molecule drugs

The Connectivity Map (CMAP) database (http://www.broadinstitute.org) could be beneficial for researchers in the identification of probable molecular drugs closely associated with diseases, including cancer. The enrichment scores were -1–1, with a negative score showing that BLCA patients could benefit from this drug.

### 2.6 Construction and validation of the prognostic signature of ROS

The prognosis-associated ROS-related genes were identified via Univariate Cox regression (UCR) analysis (survival package) and least absolute shrinkage and selection operator (LASSO) regression analysis (glmnet and survival package) with *P* < 0.05 in the TCGA dataset, followed by incorporating the results into the Multivariate Cox Regression (MCR) analysis. Finally, a ROS-correlated gene signature related, to OS was constructed based on MCR analysis results. The Risk Score (RS) was generated by this formula: RS = (Coef1*expression mRNA1) + (Coef2*expression mRNA2) + (Coef n * expression mRNA n), where Coef represents the MCR model coefficient of relevant mRNA. Based on the RS mean, patients were classified into high-risk group (HRG) and low-risk group (LRG), employing the Kaplan-Meier (K-M) method to compare the survival outcomes between different groups. Our study deployed time-related ROC analysis to determine the predictive prognostic value of the PS. Both T-distributed stochastic neighbor embedding (t-SNE) analysis alongside principal component analysis (PCA) were implemented to examine the risk signature classification capacity with the R packages “Rtsne” and “ggplot2”, employing the same approach to calculate the RS and then validated the ROS-related gene signature in the GSE13507 dataset.

### 2.7 Development of a nomogram

We explored the relationships between the PS and clinical features (age, sex, T/N/TNM stages, and tumor grade) in the TCGA dataset via the chi-squared test. Then, stratified analysis was performed to further examine the PS reliability and stability of ROS in the prediction of BLCA patients’ OS. Additionally, we implemented UCR and MCR analyses to explore whether the RS was of independent prognostic value. Both RS and clinical features were incorporated to establish an OS-related nomogram, estimating the nomogram’s predictive capability by generating a calibration curve.

### 2.8 Gene set enrichment analysis and immune cell infiltration and tumor mutational burden analyses

GSEA was implemented to investigate the latent mechanisms among different groups based on GSEA software (version 4.1.0). Then, we acquired mutation information for BLCA patients by accessing the TCGA database, calculated the total mutation number for each sample, and analyzed the top mutational genes among different risk groups using the maftools package. The TIMER, CIBERSORT, CIBERSORT-ABS, XCELL, QUANTISEQ, EPIC, and MCP-counter methods were utilized for the analysis of the ICI levels of 22 distinct leukocyte subsets in both groups. *P* < 0.05 indicated statistically significant.

### 2.9 Chemotherapeutic response analysis

The Genomics of Drug Sensitivity in Cancer (GDSC, http://www.cancerrxgene.org) database was accessed to predict BLCA patients’ response in both groups to chemotherapy drugs. Eventually, we assessed chemosensitivity by calculating the half-maximal inhibitory concentration (IC50) through the R package pRRophetic, with *P* < 0.05 indicating statistical significance.

### 2.10 Patient sample

Between 2022 and 2024, 20 BLCA tissue and their corresponding non-tumor tissue specimens were collected from Shaoxing People’s Hospital for immunohistochemical staining (IHC) and western blot. No patient in this study had received radiation therapy, adjuvant therapy, or preoperative chemotherapy. The samples from Shaoxing People’s Hospital were collected with informed consent, and the use of the stored cancer specimens and clinical data was granted clearance by the Academic Ethical Committee of Shaoxing People’s Hospital (ethical approval number: 2022-K-Y-054-01). The study was executed in a way that aligned with the Declaration of Helsinki.

### 2.11 Immunohistochemistry

IHC images of key genes in BLCA and normal tissue samples were acquired through the Human Protein Atlas (HPA) database while evaluating the staining intensity following the HPA database standard (https://www.proteinatlas.org/). Use anti-AKR1B1 (1:1000; Proteintech,15439-1-AP). Rabbit monoclonal antibody was used for immunohistochemistry of paraffin-embedded human and nude mouse BLCA specimens. In short, samples were processed using dewaxing, hydration, antigen extraction, IHC labeling, and pathology scores.

### 2.12 Cell culture, treatments, and siRNA transfection

Human BLCA cells (T24 and 5637) were procured from Procell Life Science & Technology Company (Hubei, China). Herein, we grouped the logarithmic growth phase cells into the control, siAKR1B1-negative control (NC), and siAKR1B1 groups. The two cell lines were cultured in MCCOY’S 5A (Gibco, USA) and 1640 (Gibco, USA) medium supplemented with 10% fetal bovine serum (FBS, Gibco, USA) and 1% penicillin/streptomycin at 37°C and 5% CO_2_. The cells went through treatment with 5 µl of siAKR1B1, using Lipofectamine 2000 to dilute the solution in Opti-MEM for 5 min. The solution was thereafter mixed and allowed to incubate at ambient temperature for a duration of 20 min, followed by introducing the composite into the cell culture plate. After a 48-h period of transfection, the cells were gathered for additional assessments.

### 2.13 Western blot analysis

Proteins were subjected to extraction using RIPA buffers and quantification by BCA kits. The protein eluate went through separation utilizing 10% SDS-PAGE and transferred to a PVDF membrane that was blocked and then incubated with primary and secondary antibodies. AKR1B1 (Proteintech, 15439-1-AP) and β-catenin (Abcam, ab32572) were detected by imaging with enhanced chemiluminescence reagents (Merck Millipore, Billerica, MA).

### 2.14 CCK8 assay

100 μL of suspension containing 5000 transfected cells was dispensed into each well of a 96-well plate along with 10 μL of CCK8 solution (MCE, HY-K0301). The plate was then placed in a cell culture incubator for 1 hour, following which absorbance readings were taken at 450 nm and recorded.

### 2.15 Colony formation assay

3600 BLCA cells were equally distributed into six-well plates and incubated at 37°C with 5% CO_2_ for 14 days with regular medium changes. Post-incubation, the cells were fixed and stained using 4% paraformaldehyde and 0.1% crystal violet for 20 minutes each, after which images were captured and data documented.

### 2.16 Edu assay

5-ethynyl-2′-deoxyuridine (EdU) assay kit (MCE, China) was used as instructed by the manufacturer. In this experiment, BLCA cells were cultured in 96-well plates, with a seeding density of 4,000 cells per well, after incubation at 37°C for 72 hours. Next, BLCA cells were exposed to 10 μM EdU for 2 hours at 37°C. Subsequently, the cells were fixed using 4% paraformaldehyde and permeabilized with 0.5% Triton X-100 for 15 minutes at room temperature. After removing the fixatives, the cells were washed with PBS containing 1% BSA. Lastly, the cells were incubated in Click Additive Solution, protected from light, for 30 minutes and then stained with Hoechst to label the nucleus. Microscopic images were captured to observe the EdU detection samples. The proliferation of cells was further assessed by calculating the ratio of EdU-positive cells to the overall cell count.

### 2.17 Transwell assay

The transfected HOS and 143B cell lines were cultured with the serum-free DMEM and serum-free 1640, respectively, in a Transwell upper chamber. Corresponding culture medium containing 10% FBS was added to the lower chamber. The cells were incubated at 37°C with 5% CO2 for 48 hours, fixed with formaldehyde, stained with crystal violet, and visualized under a microscope for analysis.

### 2.18 Statistical analysis

Statistical analysis was conducted using the R software (version 4.0.5). The significance of differential gene expression was ascertained using adjusted p-value to correct for the multiple testing phenomenon, with a significance threshold set at p-value < 0.05. Another statistical analysis was conducted with SPSS Statistics software version 20. The values were compared by one-way ANOVA or independent-samples Student’s t test. Statistical significance was determined at *p < 0.05, **p < 0.01, or ***p < 0.001. Values are presented as the mean ± SEM. Error bars indicate the SEM unless otherwise noted.

## 3 Results

### 3.1 Identification of ROS-related genes in BLCA

**Supplementary Figure 1** illustrates the workflow diagram of this study. In total, we obtained 1749 ROS-related genes with relevance scores > 0.5 from the Gene Cards database and 70 ROS-related genes from the MSigDB database, acquiring 1767 genes after removing the overlapping genes. However, from the 1,767 genes, we eventually extracted the expression profiles of 1,719 ROS-associated genes identical to those in 412 and 19 BLCA and normal bladder tissue samples, respectively, in the TCGA dataset. By applying cutoff criteria of FDR < 0.05 and |log2 FC| > 1, 308 ROS-related Differentially Expressed Genes (DEGs) were identified; of them, 138 were downregulated, and 170 were upregulated. Moreover, GO, KEGG, and PPI analyses were deployed to explore the possible roles of ROS-associated genes. Both Univariate Cox regression (UCR) and LASSO analyses were performed to screen for prognostic ROS-related genes, and 71 genes were included in subsequent analyses (p<0.05). As a means to guarantee the clinical outcomes’ stability and reliability based on the 71 genes, we conducted LASSO analysis to further screen for prognostic ROS-related genes, identifying 31 genes related to OS. The MCR analysis identified 17 ROS-related genes (JUN, CALR, P4HB, ELN, MYC, FASN, REV3L, VHL, NID1, SLC38A1, TFRC, AKR1B1, ITGA3, CGB5, HLA-G, FADS1, and ORM1) that were utilized to construct a PS, which was subsequently validated in the GSE13507 cohort. Both K–M survival and time-dependent receiver operating characteristic (ROC) curves were employed to evaluate the prognostic value of the PS. A nomogram was constructed, aiming at predicting the outcomes of BLCA patients in combination with the PS and clinical factors. GSEA, ICI, and TMB analysis were implemented for the exploration of the molecular characteristics of the PS. The GDSC database was accessed for the prediction of chemotherapy response according to the PS. Candidate SMDs targeting ROS-related genes were screened against the CMAP database. To verify the authenticity of the data, the PS protein expression levels were detected through the HPA. AKR1B1 was selected for *in vitro* experimental validation, demonstrating the reliability of the ROS-related genes we constructed.

### 3.2 Functional assays of the selected prognostic genes and Protein-protein interaction network construction

**Figure 1:**
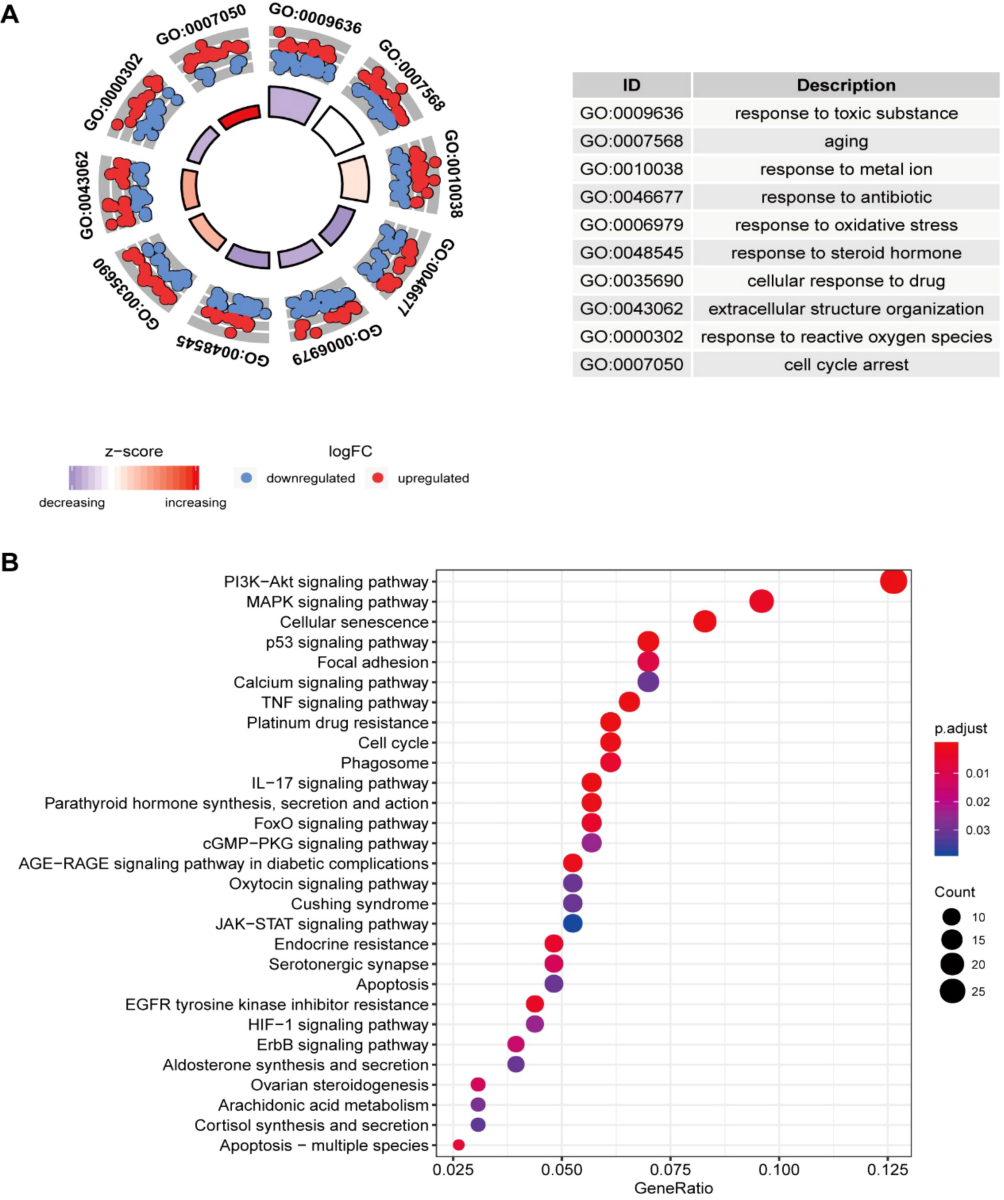
Enrichment analysis of ROS-related differentially expressed genes (DEGs) and PPI. **(A)** GO and **(B)** KEGG analyses.

**Figure 2:**
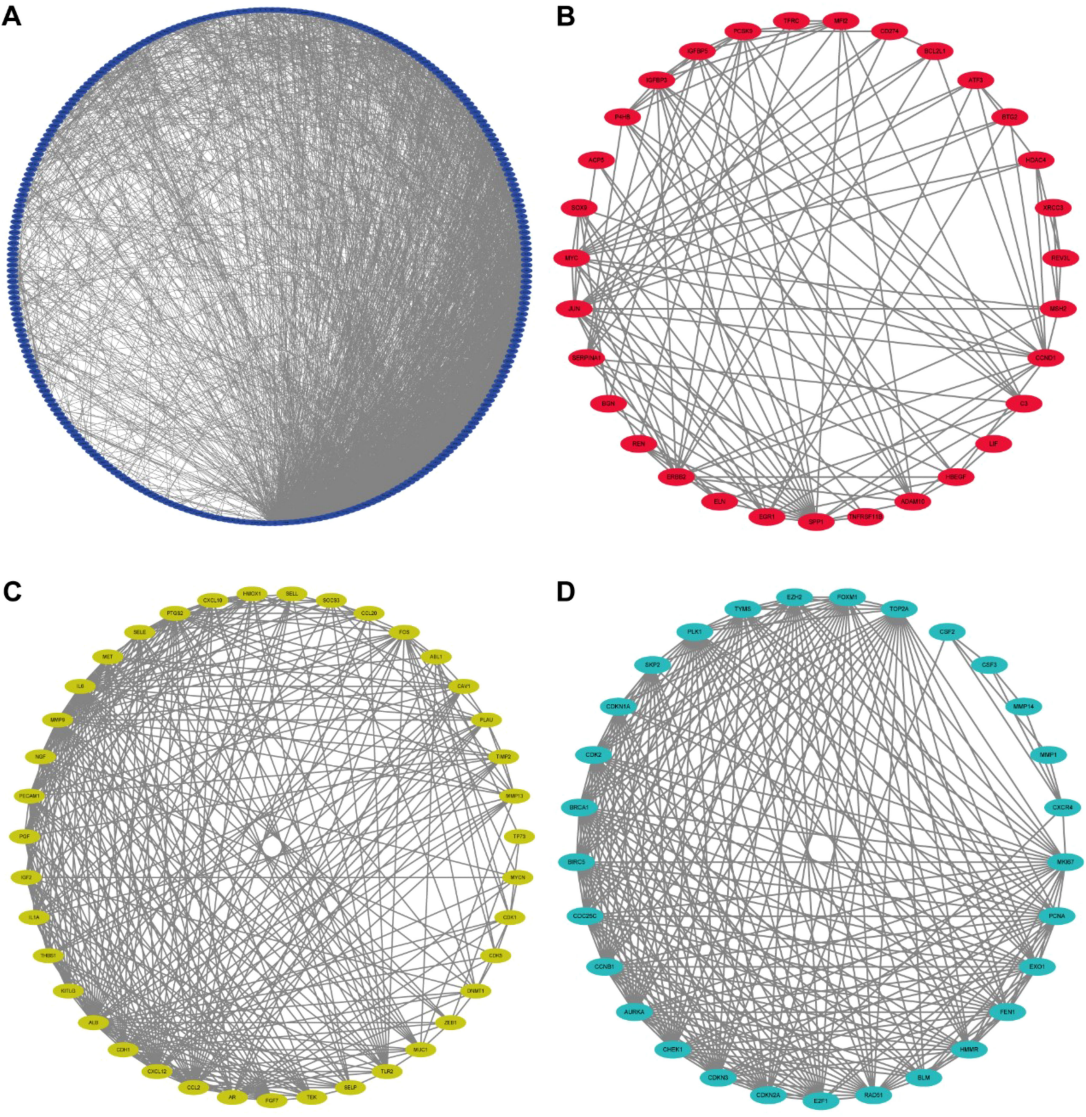
Protein-protein interaction (PPI) network. **(A)** Protein-protein interaction (PPI) network of differentially expressed ROS-related genes. **(B-D)** Key models of PPI networks.

The GO analysis findings represented that ROS-associated genes were involved in multiple biological processes, including the response to toxic substances, aging, metal ions, oxidative stress, and ROS, besides cell cycle arrest and the cellular response to drugs (**Figure 1A**). The KEGG analysis findings showcased that these genes exhibited main involvement in multiple pathways, including the p53, platinum drug resistance, cell cycle, ErbB, PI3K-Akt, TNF, cellular senescence, IL-17, MAPK, HIF-1, and cGMP-PKG signaling pathways (**Figure 1B**) (26–28). For better comprehension of the involvements of ROS-associated genes in BLCA, a PPI network was established and visualized through the utilization of STRING database and Cytoscape software, which included 298 nodes and 2859 edges (**Figure 2A**). The MCODE plugin identified three crucial modules of target genes, and the critical modules consisted of 39 nodes and 321 edges, 29 nodes and 250 edges, and 31 nodes and 123 edges (**Figures 2B–D**).

### 3.3 Small-molecule drugs

Using the CMAP database, candidate SMDs for BLCA were identified based on ROS-related DEGs, identifying eleven SMDs (0297417-0002B, 5248896, puromycin, blebbistatin, anisomycin, STOCK1N-35215, methylergometrine, clofilium tosylate, verteporfin, withaferin A, and rottlerin) with anticancer functions in BLCA progression with enrichment scores < -0.8, *p* < 0.01, and n > 2 as the screening criteria (**Table 1**).

**Table 1:** The 11 small molecule drugs of CMP dataset analyses results.

cmap name	mean	n	enrichment	*p*-value	percent non-null
0297417-0002B	-0.779	3	-0.979	0.00004	100
puromycin	-0.765	4	-0.952	0	100
5248896	-0.668	2	-0.948	0.00594	100
blebbistatin	-0.679	2	-0.936	0.00861	100
anisomycin	-0.662	4	-0.933	0.00002	100
STOCK1N-35215	-0.691	3	-0.926	0.00062	100
methylergometrine	-0.64	4	-0.863	0.00064	100
verteporfin	-0.607	3	-0.844	0.00757	100
rottlerin	-0.68	3	-0.84	0.00817	100
withaferin A	-0.569	4	-0.832	0.00145	100
clofilium tosylate	-0.597	3	-0.832	0.00937	100

### 3.4 Construction and validation of the ROS-based prognostic signature

**Figure 3:**
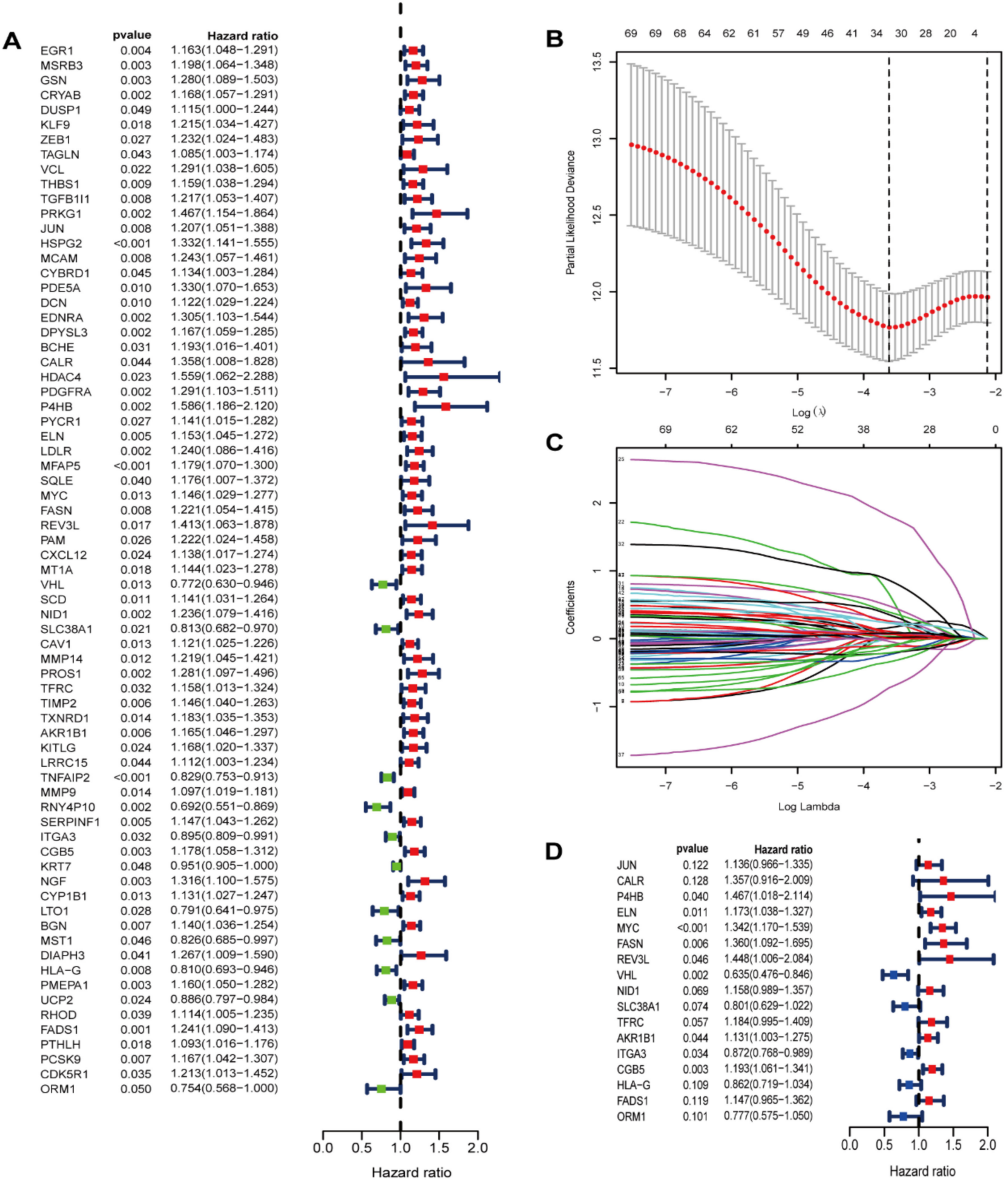
Identification of prognostic ROS-related genes in TCGA dataset. **(A)** Screening prognostic ROS-associated genes through univariate Cox regression analysis; **(B)** Incorporating the prognostic ROS-associated genes into the LASSO regression analysis; **(C)**The prognostic ROS-related genes were incorporated into the LASSO regression analysis. **(D)** Screening prognostic ROS-related genes through multivariate Cox regression analysis.

**Figure 4:**
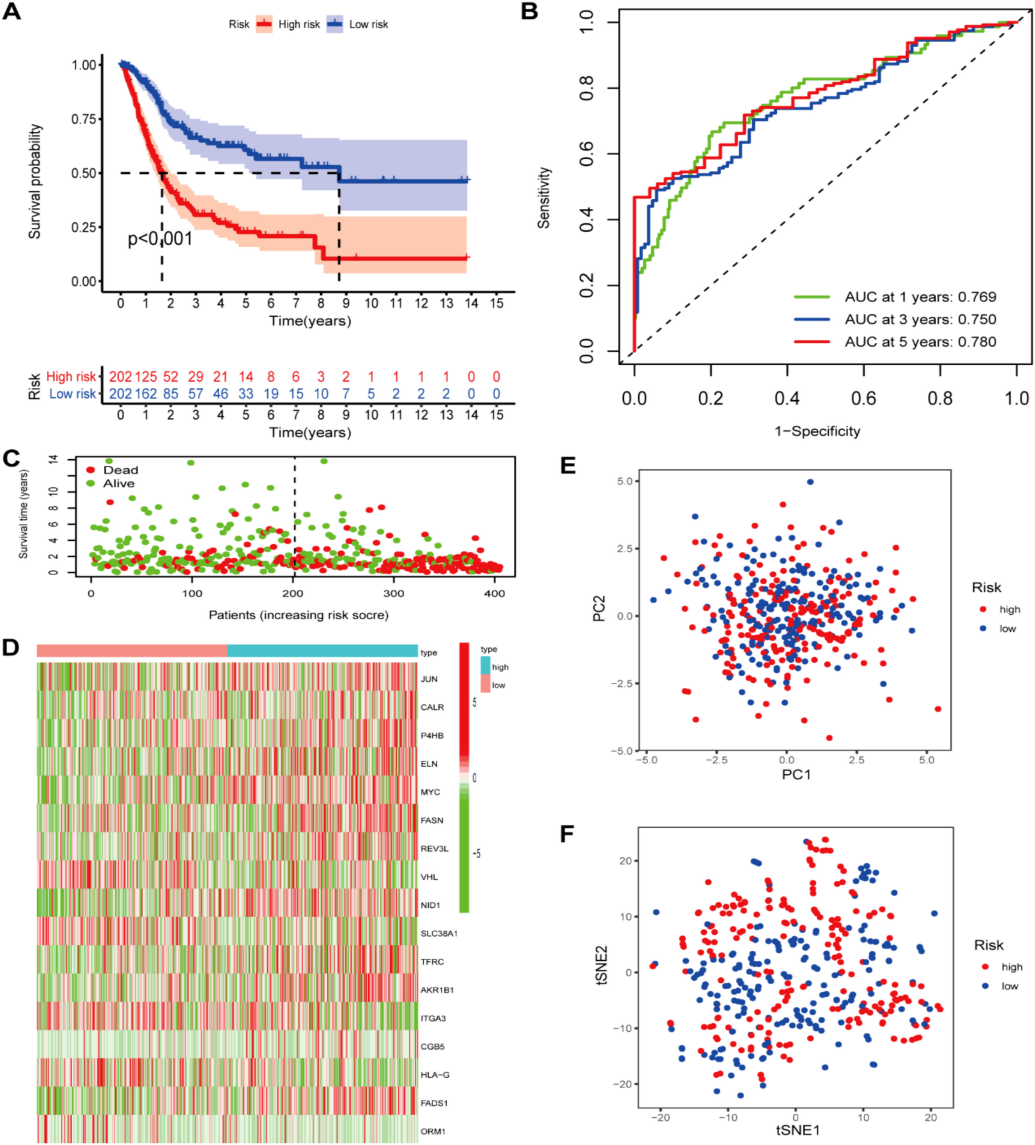
Prognostic ROS-based signature construction in TCGA dataset. **(A)** Kaplan-Meier survival analysis of BLCA patients between different groups; **(B)** Survival status distribution relying on the median risk score; **(C)**Time-independent ROC analysis of 5-year survival risk scores; **(D)** Heatmap showing the differences of 17 ROS-related genes between different groups. **(E)** PCA analysis; **(F)** t-SNE analysis.

Our study conducted UCR analysis to identify ROS-related DEGs notably correlated with OS, and 71 genes were included in subsequent analyses (*p*<0.05) (**Figure 3A**). Aiming to ensure the clinical outcomes stability and reliability based on the 71 genes, LASSO analysis was conducted to further screen for prognostic ROS-related genes, and we identified 31 genes related to OS (**Figures 3B, C**). Multivariate Cox Regression (MCR) analysis identified 17 ROS-related genes (JUN, CALR, P4HB, ELN, MYC, FASN, REV3L, VHL, NID1, SLC38A1, TFRC, AKR1B1, ITGA3, CGB5, HLA-G, FADS1, and ORM1) that were used to construct a PS (**Figure 3D**). A ROS-based RS was established depending on the coefficient of 17 genes according to this formula: risk score = (0.3078 × FASN expression) + (0.305 × CALR expression) + (0.3832 × P4HB expression) + (0.1599 × ELN expression) + (0.2941 × MYC expression) + (0.3702 × REV3L expression) + (-0.4548 × VHL expression) +(0.147 × NID1 expression) + (-0.2213 × SLC38A1 expression) + (0.1687 × TFRC expression) +(0.123 × AKR1B1 expression) + (-0.1371 × ITGA3 expression) +(0.1762 × CGB5 expression) + (-0.1483 × HLA-G expression) +(0.1368 × FADS1 expression) + (-0.252 × ORM1 expression) + (0.1274 × JUN expression). Subsequently, we classified patients into HRG and LRG in accordance with the median RS. The LRG patients had longer OS than those in the HRG (p < 0.05) (**Figures 4A, C**). Time‐dependent ROC analysis depicted that the signature AUC in the TCGA cohort was 0.78 at 5 years (**Figure 4B**). A heatmap was generated to show the differences in 17 ROS-related genes between the different groups (**Figure 4D**). PCA and t-SNE analyses indicated the signature’s good classification ability (**Figures 4E, F**). Additionally, the prognostic capacity of our constructed PS was validated in the GSE13507 dataset. The results in GSE13507 (**Supplementary Figure 2**) were consistent with previous results, which demonstrated the good performance of the PS in predicting OS.

### 3.5 Establishment and validation of the risk scores model

**Figure 5:**
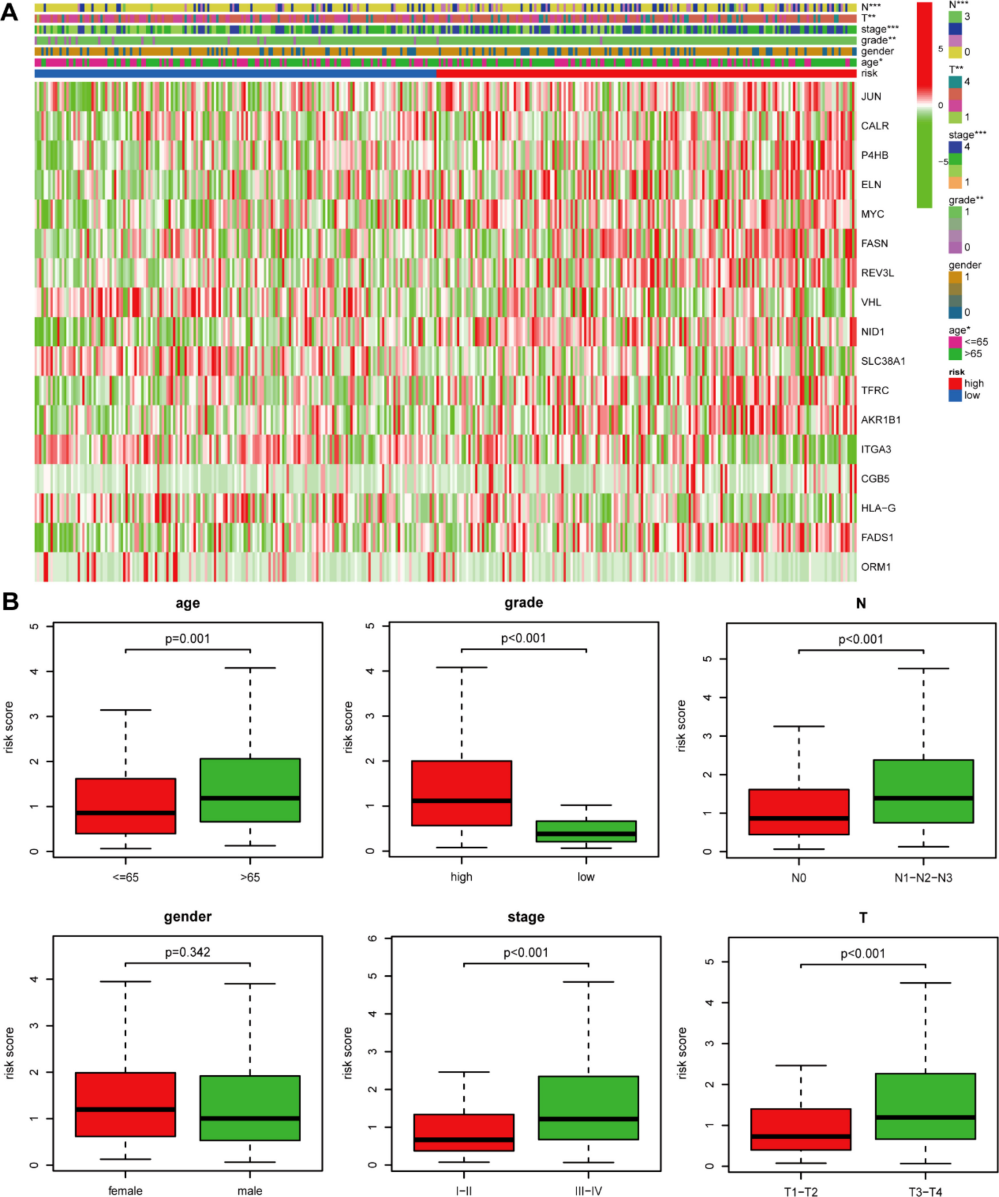
The risk score and clinicopathological factor correlation in the TCGA dataset. **(A)** The heatmap (*: 0.01<*P*<0.05; **: 0.001<*P*<0.01; ***: *P*<0.001) and **(B)** Boxplot show the risk score and clinicopathological factor correlation.

**Figure 6:**
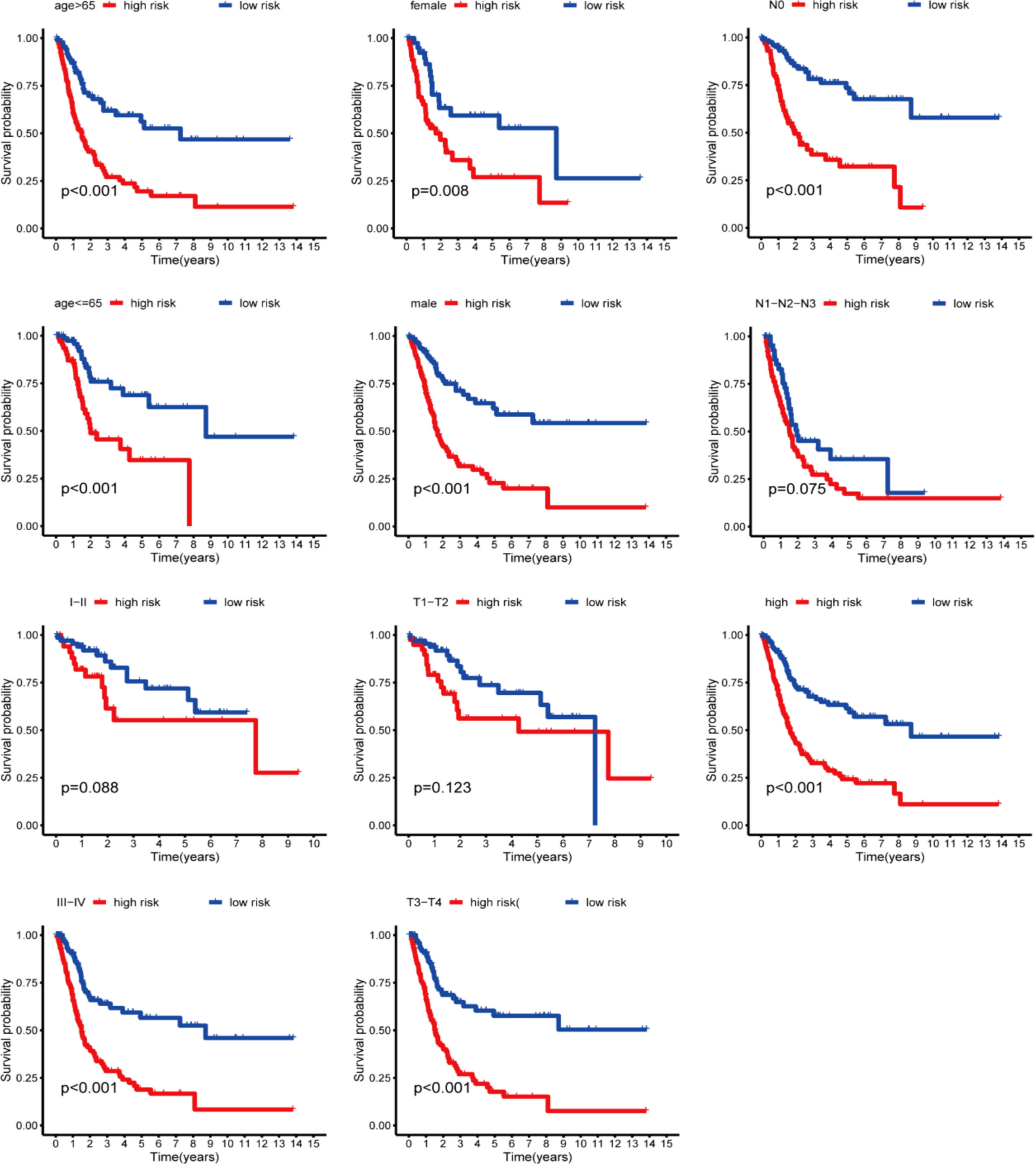
Kaplan-Meier curves stratification of OS by gender, age, grade, or N/T/TNM stages between both risk groups.

**Figure 7:**
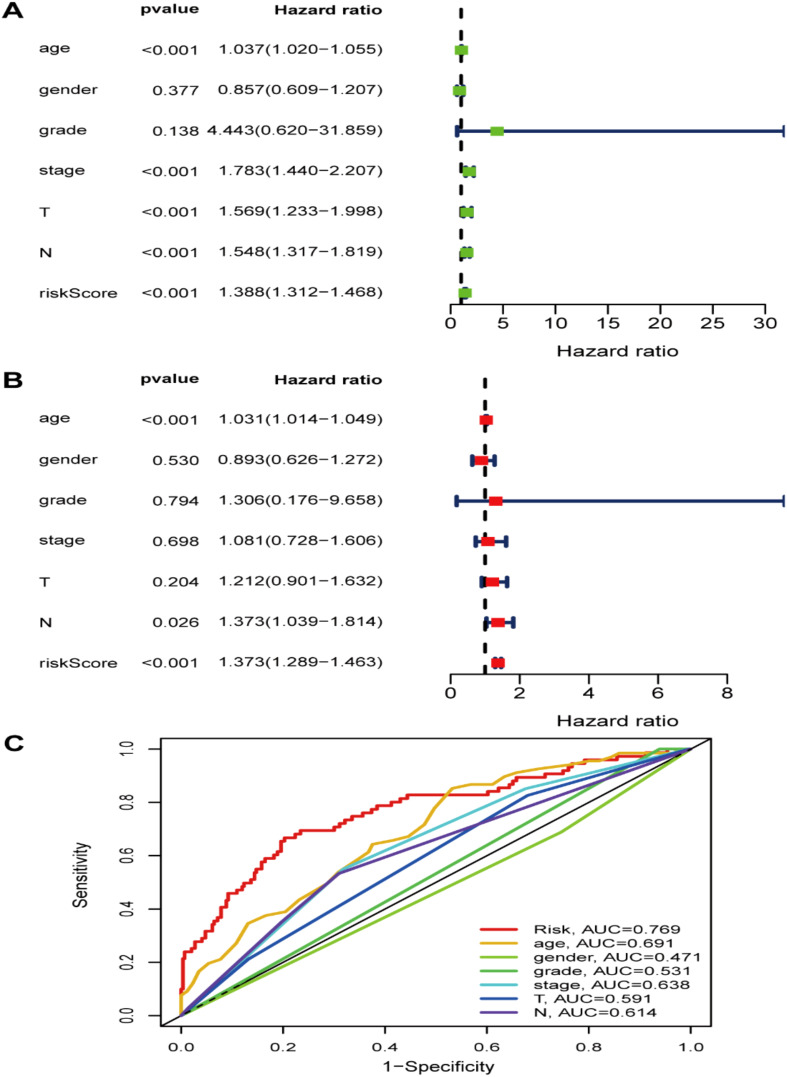
The risk signature as an independent BLCA prognostic factor in the TCGA dataset. **(A)** The OS risk score and clinicopathological factor correlations by univariate and **(B)** multivariate Cox regression analysis. **(C)** ROC curves of the clinical characteristics and risk score.

**Figure 8:**
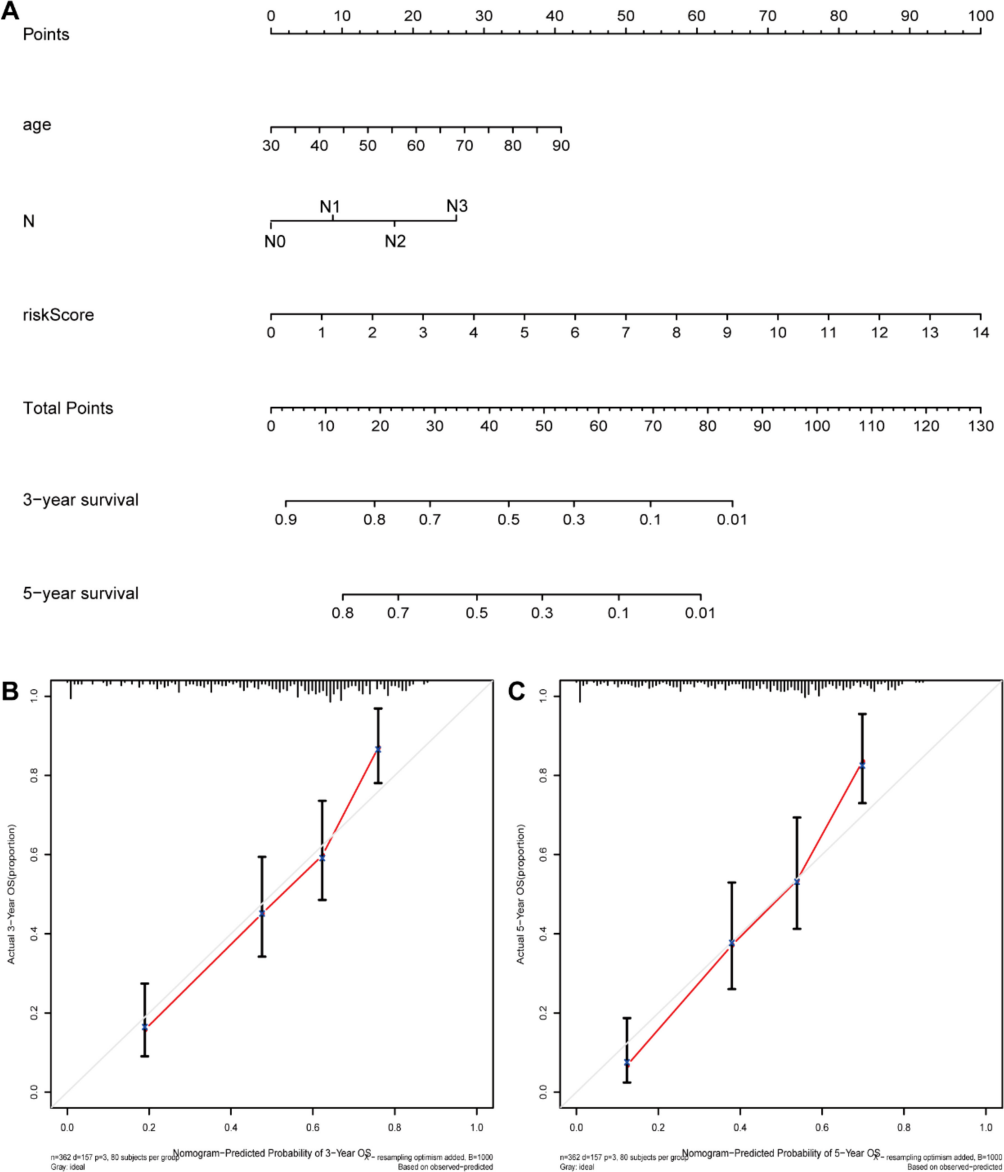
The nomogram construction. **(A)** Nomogram predicting 3‐ or 5‐year OS. **(B)** Calibration plots predicting 3‐ and **(C)** 5‐year OS.

We first compared the ROS-based RSs among various subgroups classified by clinicopathological characteristics (TNM stage, sex, grade, age, T stage, and N stage). The RSs exhibited a significant correlation with clinicopathological factors and were markedly elevated in the following subgroups: >65 years of age, advanced T stage (T3/4), N stage (N1/2/3), pathological grade (High), and TNM stage (Stage III-IV) (**Figure 5**). Subsequently, stratification analysis was conducted relying upon the clinical characteristics (age, sex, grade, and TNM/T/N stages). Male or female sex, age (>65 years) or (<=65 years), T stage (T3-T4), N stage (N0), pathological grade (High), and TNM stage (Stage III–IV) were associated with inferior OS in the high-risk subgroup (*P* < 0.05) (**Supplementary Figure 2**), with no difference in OS in the T stage (T1/2), N stage (N1/2/3), or TNM stage (Stage I-II) subgroup (**Figure 6**). Furthermore, to evaluate whether the RS was an autocephalous prognostic indicator for BLCA patients, univariate and multivariate Cox proportional hazard models were implemented. According to UCR analysis results, age, TNM/T/N stages, and RS were related to unfavorable OS (**Figure 7A**). According to the multivariate analysis, age, N stage, and RS were still associated with unfavorable OS (**Figure 7B**). Multiparameter ROC curve analyses also revealed that the AUC of the RS was 0.769 (**Figure 7C**), indicating that compared with traditional clinical prognostic indicators, the ROS-based RS exhibited remarkable performance in predicting prognosis. Collectively, the ROS-based RS was an autocephalous prognostic factor. A nomogram including RS, age, and N stage we established to predict the outcomes of BLCA patients (**Figure 8A**), with the calibration curve elucidating the nomogram’s good performance in predicting patient prognosis (**Figures 8B, C**).

### 3.6 GSEA

**Figure 9:**
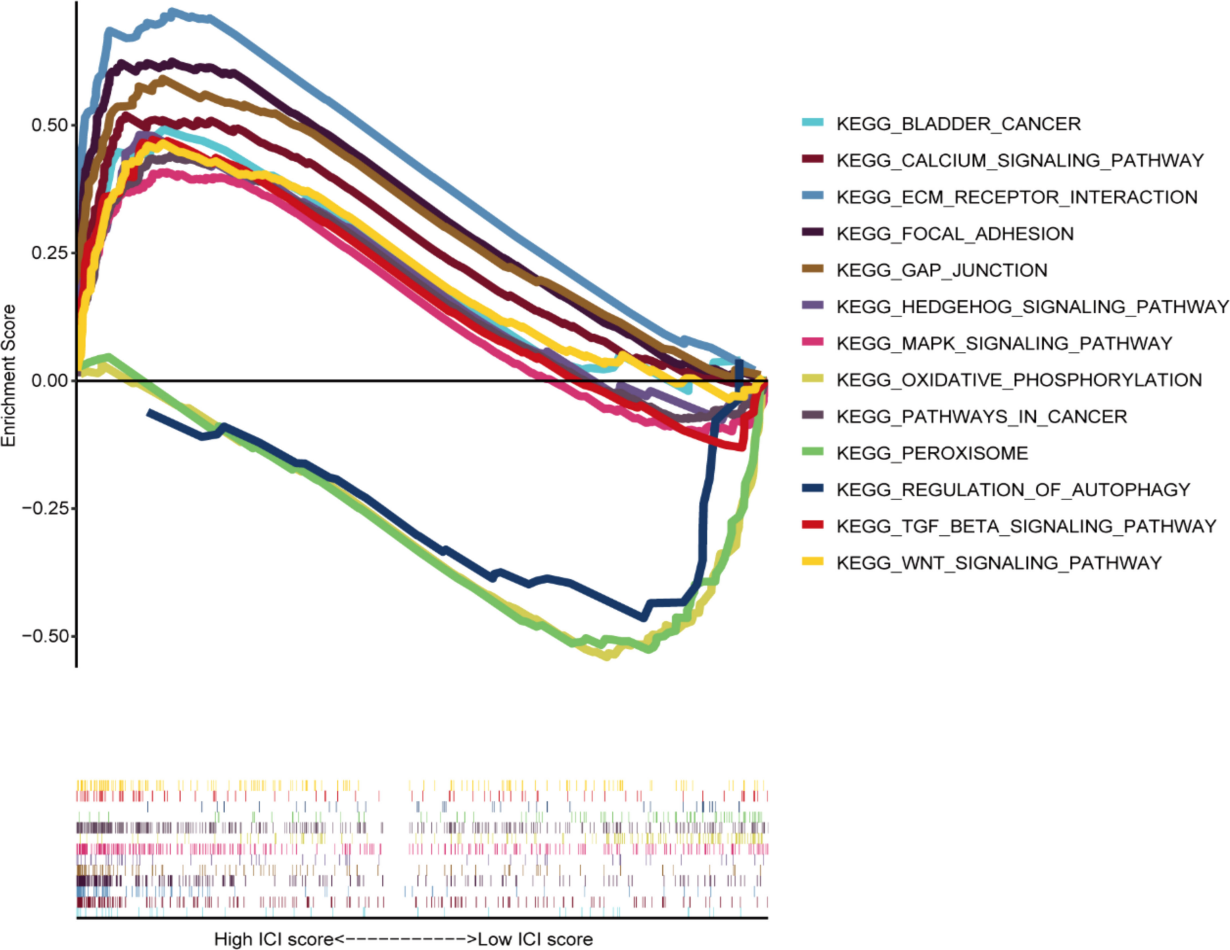
Gene set enrichment analysis among different groups.

The GSEA results demonstrated that carcinogenic signaling pathways, such as calcium, focal adhesion, ECM receptor interaction, MAPK, BLCA, GAP junction, Wnt, Hedgehog, cancer, and TGF-β signaling pathways, exhibited main enrichment in the HRG (**Figure 9**). Several metabolism-associated signaling pathways, including autophagy regulation, peroxisomes, and oxidative phosphorylation, were highly enriched in the LRG.

### 3.7 Immune cell infiltration

**Figure 10:**
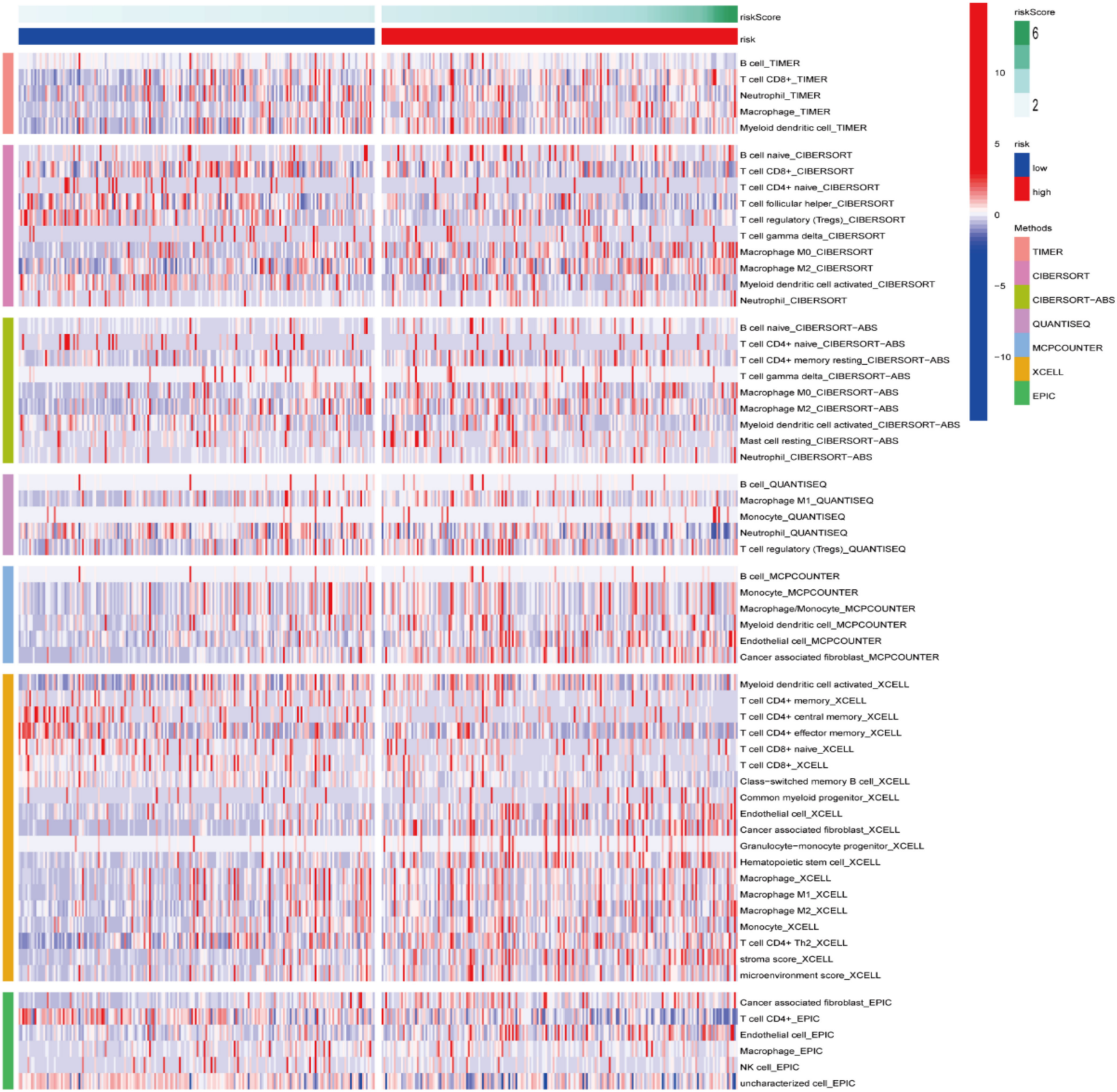
Immune cell infiltration between both risk groups.

A heatmap of the ICI data obtained via CIBERSORT, MCPcounter, XCELL, TIMER, CIBERSORT-ABS, QUANTISEQ, and EPIC analyses (**Figure 10**) suggested that the RS was correlated with ICI in BLCA. Additionally, significant differences were observed in the fractions of distinct leukocyte subsets between both groups. The proportions of naive B cells and M0/M2 macrophages were lower in the HRG, whereas the proportions of CD8+/CD4+T cells/were greater in the LRG.

### 3.8 Tumor mutational burden analysis

**Figure 11:**
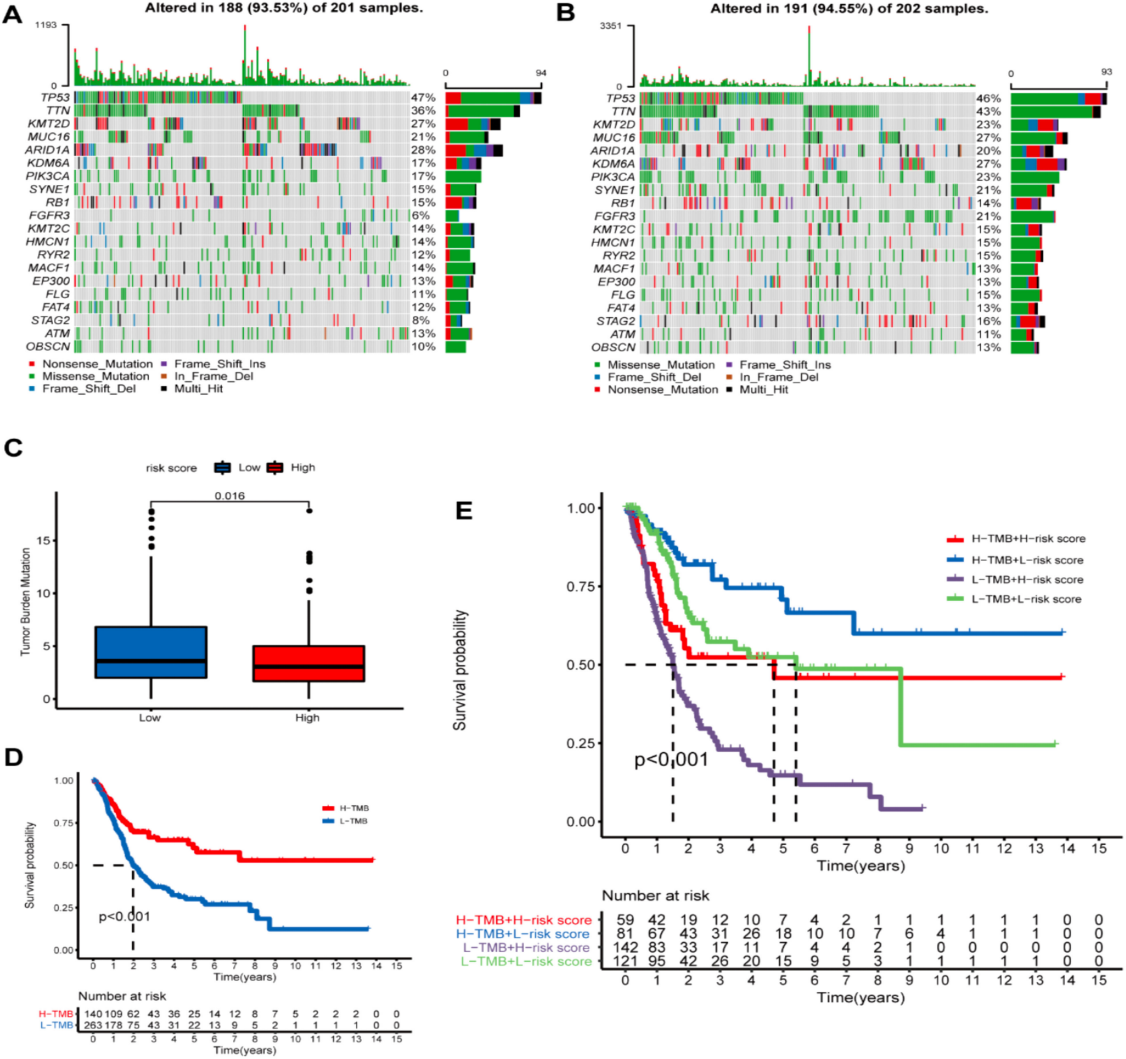
Tumor mutational burden (TMD) analysis. **(A)** Demonstrating the top 20 mutational genes within the high- and **(B)** low-risk groups. **(C)** TMB difference in both risk groups. **(D)** Kaplan-Meier (K-M) survival analysis of BLCA patients with high or low TMB. **(E)** K-M curve analysis stratification of OS by TMB and the prognostic signature.

The mutation profile results among different risk groups in the TCGA dataset revealed somatic mutations in 93.53% (118) and 94.55% (191) of the BLCA patients in the HRG (**Figure 11A**) and LRG (**Figure 11B**), respectively. TP53, TTN, KMT2D, MUC16, ARID1A, KDM6A, PIK3CA, SYNE1, RB1, and KMT2C were the top 10 mutated genes in the HRG. TP53, TTN, KMT2D, MUC16, ARID1A, KDM6A, PIK3CA, SYNE1, RB1, and FGFR3 were the top 10 mutated genes in the LRG. Furthermore, the proportions of somatic mutations in KDM6A and FGFR3 significantly differed between both groups. Additionally, the LRG patients had more mutation events than the HRG (**Figure 11C**). Patients having a high TMB appeared to possess a better prognosis than those with a low TMB (**Figure 11D**). Further, we investigated the collaborative interaction effect of the ROS-based RS and TMB on prognosis (**Figure 11E**). We found that the HRG patients having a high TMB had shorter OS than those in the LRG with a high TMB, and the LRG patients having a low TMB had longer OS than those in the HRG with a low TMB. Interestingly, patients having a high TMB exhibited better OS than those having a low TMB in the HRG, and patients having a low TMB displayed worse OS than those having a high TMB in the LRG. Patients possessing high TMB in the LRG had a greater OS than patients in the other three patient groups, and patients having low TMB in the HRG tended to have a significantly worse OS than patients in the other three patient groups. Collectively, the ROS-based RS might be a probable biomarker for predicting OS in BLCA patients.

### 3.9 Chemotherapeutic response analysis

**Figure 12:**
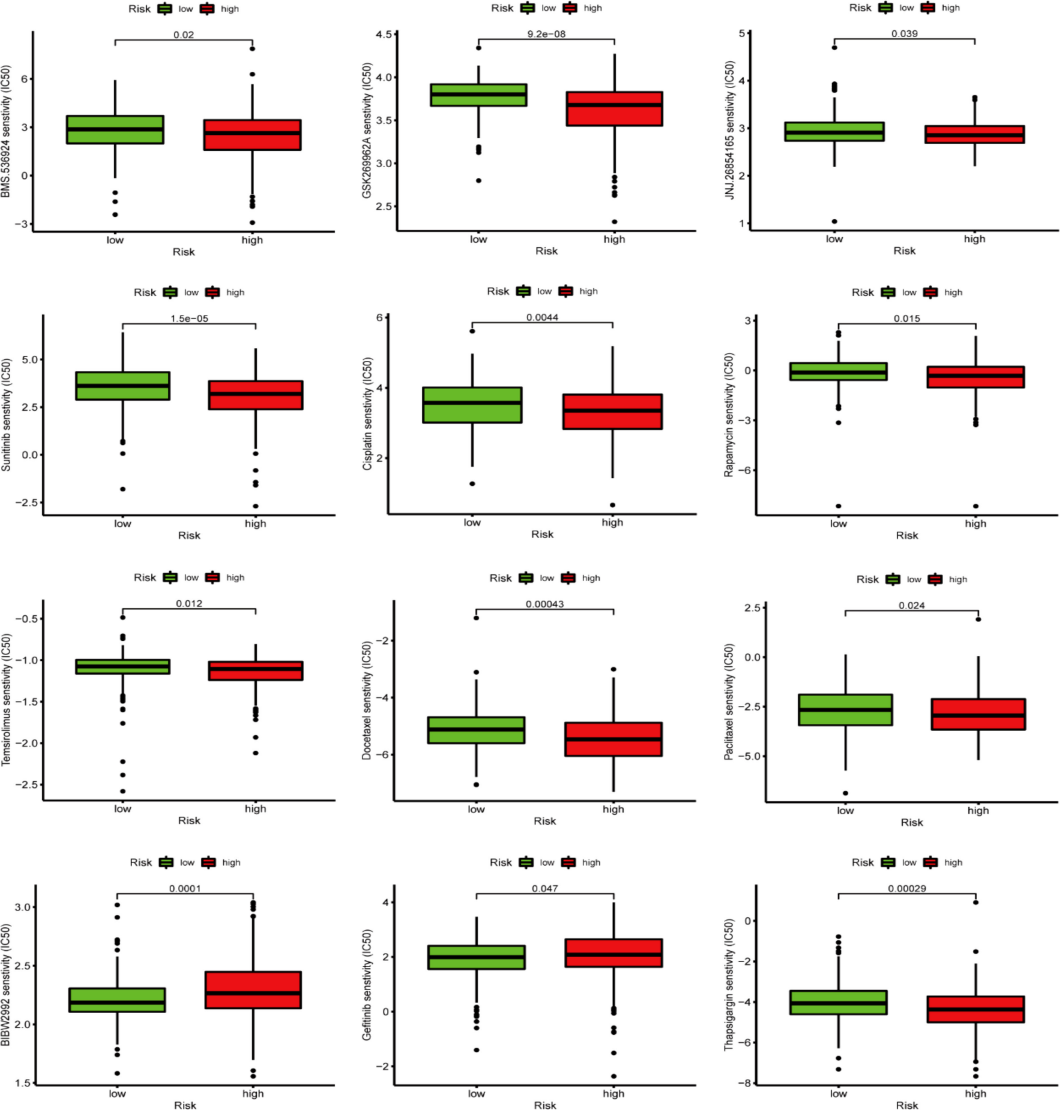
GDSC database-based chemotherapy response prediction.

The GDSC database analysis findings depicted that the IC50 values of chemotherapy drugs, including GSK269962A, BMS.536924, JNJ.26854165, docetaxel, temsirolimus, cisplatin, thapsigargin, sunitinib, rapamycin, and paclitaxel, were greater in LRG patients than in those the HRG. In comparison, the IC50 values of BIBW2992 and gefitinib were greater in HRG patients than in those at LRG (**Figure 12**).

### 3.10 Expression analysis of nine genes in the Human Protein Atlas database

**Figure 13:**
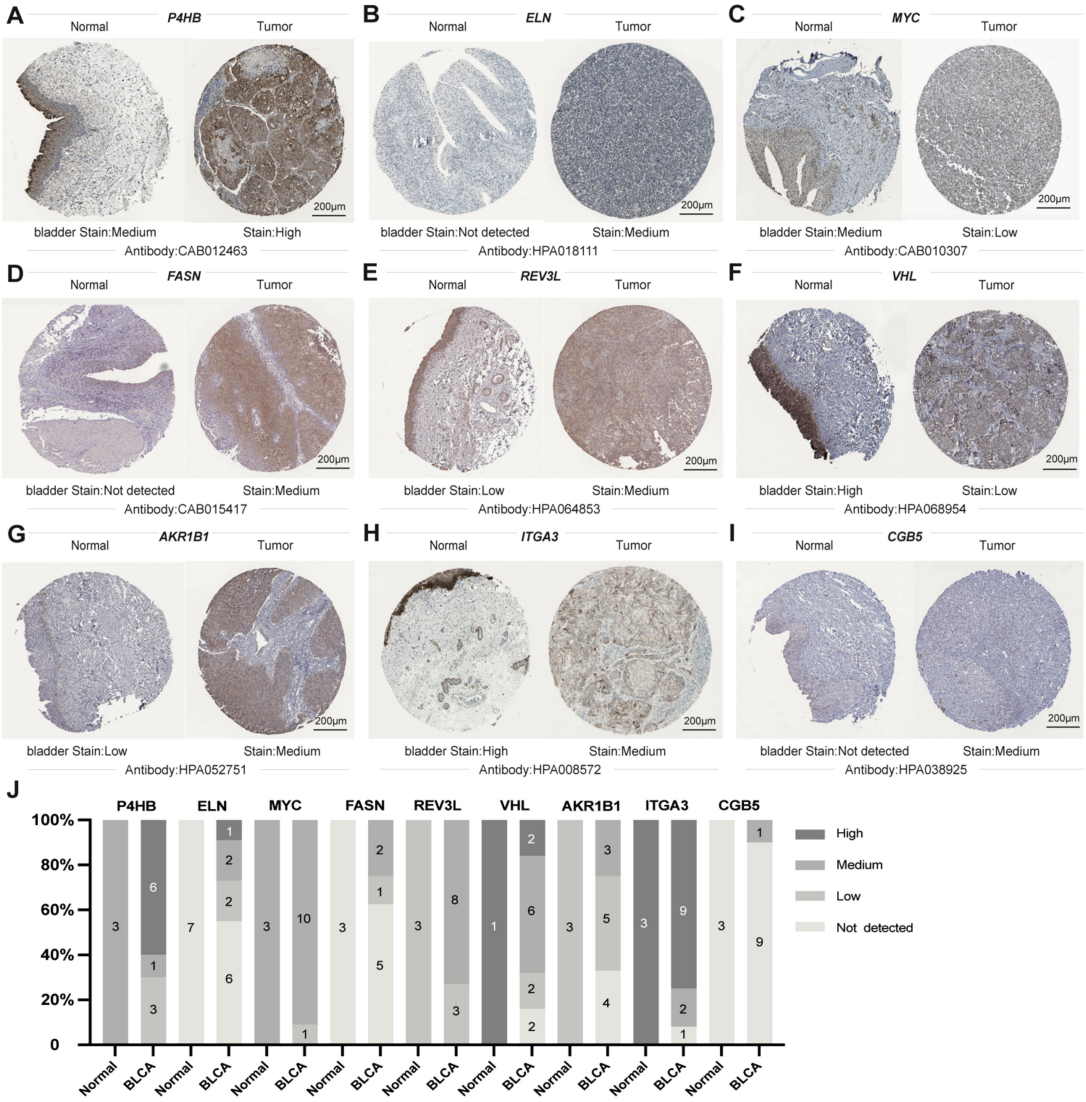
Sectional images of the differential expression of the above genes from the Human Protein Atlas. **(A–I)** representative images of P4HB **(A)**, ELN **(B)**, MYC **(C)**, FASN **(D)**, REV3L **(E)**, VHL **(F)**, AKR1B1 **(G)**, ITGA3 **(H)**, and CGB5 **(I)** protein expression from HPA databases. **(J)** Genes from HPA databases Statistical Column Stacked Plots of Characterized Protein Expression. Scale bar: 200μm.

IHC was utilized to additionally investigate the nine gene protein expression in the HPA database between BLCA and normal control tissues (**Figure 13**). In line with the RNA sequencing data, P4BH, FASN, AKR1B1, and CBG5 proteins, which have a high prognostic risk, were upregulated in tumor tissues, and MYC proteins, which have a low prognostic risk, were downregulated in tumor tissues compared with normal controls.

### 3.11 AKR1B1 affected BLCA cell viability, migration, and proliferation

**Figure 14:**
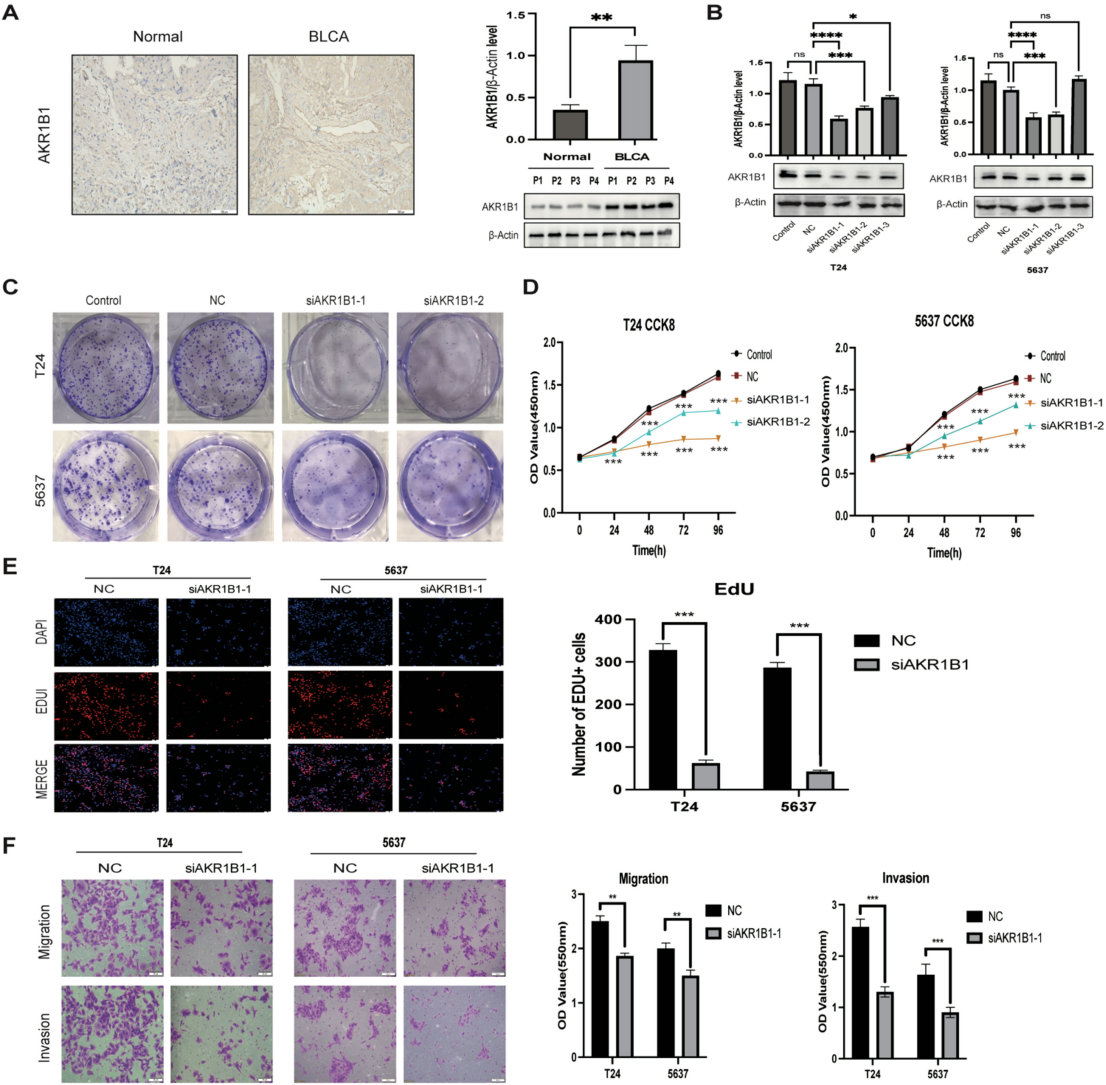
**(A)** IHC representation chart and western blot (WB) showed AKR1B1 expression in normal bladder tissue and BLCA tissue. Scale bar: 100μm. **(B)** WB detection of AKR1B1 relative expression in control, NC, and siAKR1B1 groups. **(C)** Colony formation experiment results with AKR1B1 expression. **(D)** Results of silencing AKR1B1 expression at different time points of CCK-8:24, 48, 72, 96h. **(E)** Edu assay showing proliferating cells (T24 and 5637); Edu (red) and DAPI (blue) staining. Scale bar: 50μm. **(F)** Transwell assay results in control, NC, and siAKR1B1 groups. Scale bar: 100μm. *p < 0.05, **p < 0.01, ***p < 0.001, ****p < 0.0001, ns *p* > 0.05.

While AKR1B1 has been documented in other types of cancer (29–31), its impact on BLCA remains unreported. Therefore, AKR1B1 was selected for further analysis. IHC and WB analyses elucidated AKR1B1 overexpression in BLCA tissues (**Figure 14A**). To understand the role of AKR1B1 in BLCA, we further investigated the effect of increased AKR1B1 levels in BLCA cell lines (T24 and 5637) via *in vitro* experiments. siRNA transfection successfully interfered with the mRNA expression of AKR1B1, which was confirmed by WB (**Figure 14B**). To further understand the effect of AKR1B1, colony formation analysis was also performed (**Figure 14C**), which showed that BLCA cell viability was significantly hindered after AKR1B1 was silenced by siRNA. The CCK-8 assay showcased that cell viability was impeded after the silencing of AKR1B1 expression (**Figure 14D**). In addition, an EdU proliferation assay showed that inhibiting AKR1B1 significantly lowered the percentage of EdU-positive BLCA cells (**Figure 14E**). To further test whether AKR1B1 affects BLCA cell metastasis, a Transwell assay was performed (**Figure 14F**), revealing that siRNA-mediated silencing of AKR1B1 inhibited BLCA cell migration and invasion.

## 4 Discussion

Reactive Oxygen Species (ROS) include hydroxyl radicals (·OH), superoxide anions radicals (·O2-), and hydrogen peroxide (H2O2), are considered a double-edged sword (32). Physiologically, ROS play a crucial role in organisms. Excessive ROS can damage proteins and DNA through oxidative damage, causing many diseases, including cancer. ROS can cause cancer cells to die in high concentrations (33, 34). However, the possible mechanisms and prognostic value of ROS-associated genes in BLCA remain indefinite. Our study systematically explored the expression patterns and correlations of ROS-associated genes with outcomes in BLCA. Furthermore, we established a prognosis-correlated novel signature relying on 17 ROS-related genes. Here, the ROS-based signature was associated with CD8+ T cells and chemotherapy responses. Eleven drugs were screened for treating BLCA patients. Our results offer novel insights into ROS involvement in BLCA development and progression.

In BLCA, 308 ROS-related genes were identified as Differentially Expressed Genes (DEGs); of them, 138 and 170 were downregulated and upregulated genes, respectively. Then, we explore the ROS-related DEGs’ functions through GO and KEGG analyses. According to GO analysis, these genes exhibited main enrichment in response to toxic substances, aging, metal ions, oxidative stress, and ROS, besides cellular response to drugs. KEGG analysis showcased that these genes were closely associated with cancer-, immune-, and drug resistance-correlated pathways, such as the p53, platinum drug resistance, PI3K-Akt, TNF, IL-17, MAPK, HIF-1, and cGMP-PKG pathways, suggesting that ROS-related genes are involved in tumorigenesis. Subsequently, according to the results of differential expression analyses, a PS consisting of 17 ROS-related genes (JUN, CALR, P4HB, ELN, MYC, FASN, REV3L, VHL, NID1, SLC38A1, TFRC, AKR1B1, ITGA3, CGB5, HLA-G, FADS1, and ORM1) was constructed and validated via LASSO and Cox regression analyses.

Among the seventeen ROS-related genes in our established signature, calreticulin (CALR), an ER protein with high Ca2+-binding activity, is crucial in maintaining cell homeostasis and initiating the anticancer immune response to immunogenic cell death (35, 36). Elevated CALR was correlated with favorable prognosis in distinct tumor types (36–39). CALR overexpression was linked to worse OS in natural-killer T-cell lymphoma patients (40). CALR silencing suppressed BLCA cell proliferation, migration, and lung metastasis (41). FASN can serve as an oncogene by regulating AKT signaling pathways in BLCA (42, 43). Overexpression of P4HB was notably associated with inferior outcomes, and knocking down P4HB impeded cell proliferation and enhanced GEM sensitivity via the PERK/eIF2α/ATF4/CHOP signaling pathways in BLCA (44). ITGA3 downregulation hindered tumor cell invasion and proliferation by regulating the FAK/PI3K/AKT pathway and epithelial-mesenchymal transition (45, 46). SLC38A1, a vital transporter of glutamine, has been implicated in tumorigenesis (47, 48). The expression of TFRC, a crucial member involved in ferroptosis, was significantly elevated in BLCA and promoted the tumorigenic phenotype of BLCA cells by inducing EMT (49). Aldo–keto reductase family 1 member B1 (AKR1B1) is closely implicated in cancer development and progression through various mechanisms, including EMT, ERK1/2, Ras, and PI3K-AKT signaling pathways (50). Additionally, ARK1B1 was also related to chemotherapeutic resistance and cancer stem cells (51, 52). REV3L is highly overexpressed in several cancers and facilitates cancer cell proliferation, metastasis, and insensitivity to cisplatin (53, 54). Elastin (ELN), a crucial member of the extracellular matrix family, has been documented to contribute to cancer cell invasion (55, 56). Orosomucoid 1 (ORM1), an essential immune system regulator in acute-phase reactions, might facilitate cancer cell immune evasion (57, 58). Nidogen1 (NID1), a vital component of the basement membrane, serves as an oncogene in several tumors (59–62). Chorionic gonadotropin beta polypeptide 5 (CGB5) can accelerate cancer growth and vasculogenic mimicry formation by activating the LHR signaling pathway (63). Jun represents a critical transcription factor implicated in various biological processes, including autophagy, proliferation, apoptosis, metastasis, and inflammation (64, 65). FADS1 silencing reduced cell growth by arresting the cell cycle in the G1 phase (66).

Our Univariate Cox regression (UCR) and Multivariate Cox Regression (MCR) analyses results demonstrated that the RS was a negative prognostic factor of OS in BLCA patients. Further, ROC analysis suggested that the RS outbalanced the conventional clinical characteristics in OS prediction of BLCA patients. Herein, BLCA patients with advanced clinical features (III-IV stage, Grade high, T3/4 stage, and N1/2/3 stage) had elevated RSs in comparison with patients with early clinical features (I-II stage, Grade low, T1/2 stage, and N0 stage). The RS was also related to age. Stratification analyses revealed that the RS could effectively predict BLCA patient outcomes in most subgroups other than subgroups (N1/2/3 stage, T1/2 stage, and I-II stage). Finally, we constructed a ROS-related nomogram to evaluate 3- and 5-year OS comprehensively. The calibration curve results implied that the nomogram showed excellent performance in predicting BLCA patient prognosis.

To deeply understand the potential mechanisms behind ROS-mediated differential outcomes in BLCA patients, we implemented GSEA analyses for different groups relying upon the ROS-based PS. The findings demonstrated that the HRG exhibited enrichment in cancer-associated pathways, including calcium, focal adhesion, ECM receptor interaction, MAPK, Wnt, Hedgehog, cancer, and TGF-β pathways, implying the existence of an immunosuppressive microenvironment. Meanwhile, the LRG genes exhibited main involvement in the regulation of autophagy, peroxisomes, and oxidative phosphorylation. Altogether, OS was inferior in the HRG patients than those in the LRG. The ICI is related to the malignant biological phenotypes and prognosis of cancer patients, which indicates that immunotherapy, particularly immune checkpoint inhibitor treatment, has become crucial for treating advanced tumors (67). CD8+ T cells are strongly correlated with the effectiveness of cancer immunotherapy (68). An elevated CD8+ T cell infiltration level indicated a superior prognosis in BLCA patients (69), aligning with our finding that the LRG patients possessed a greater CD8+ T cell proportion and favorable outcomes. Previous research has shown that patients having a high TMB appear to possess a prolonged survival time and an improved immunotherapy response (70). However, the TMB and immunotherapy response correlation remains controversial (71). Herein, the HRG patients exhibited a lower TMB and an inferior prognosis and might benefit from cisplatin, docetaxel, temsirolimus, thapsigargin, BMS.536924, GSK269962A, JNJ.26854165, sunitinib, rapamycin, and paclitaxel. Meanwhile, LRG patients might benefit from BIBW2992 and gefitinib.

Our study had various constraints. The ROS-based signature we developed and validated was generated via retrospective research and requires confirmation through a prospective trial. However, it is necessary to conduct more experimental validation to confirm the probable molecular mechanisms behind the PS in BLCA.

## 5 Conclusions

Conclusively, we conducted a thorough investigation of the possible functions and prognostic value of ROS-associated genes in BLCA through integrated bioinformatics analyses. In addition, a ROS-dependent PS we constructed and validated with the ability to predict the outcome and chemotherapy response of BLCA patients. Moreover, we constructed a nomogram including a ROS-based PS with clinical characteristics for 3- and 5-year OS, which could aid clinicians in clinical decision-making. To verify the authenticity of the data, we detected the signature protein expression levels through HPA. *In vitro*, siRNA-mediated AKR1B1 silencing impeded BLCA cell viability, migration, and proliferation, consistent with our projections and demonstrating the constructed ROS-related gene reliability.

## Data Availability

The original contributions presented in the study are included in the article/**Supplementary Material**. Further inquiries can be directed to the corresponding authors.
