# Supplementary material for: Exploration and validation of a novel reactive oxygen species–related signature for predicting the prognosis and chemotherapy response of patients with bladder cancer
Source: Front Immunol. 2024 Dec 19;15:1493528. doi: 10.3389/fimmu.2024.1493528 (PMC11693660; doi:10.3389/fimmu.2024.1493528)
Supplement: Supplementary file 1 [file DataSheet1.docx]

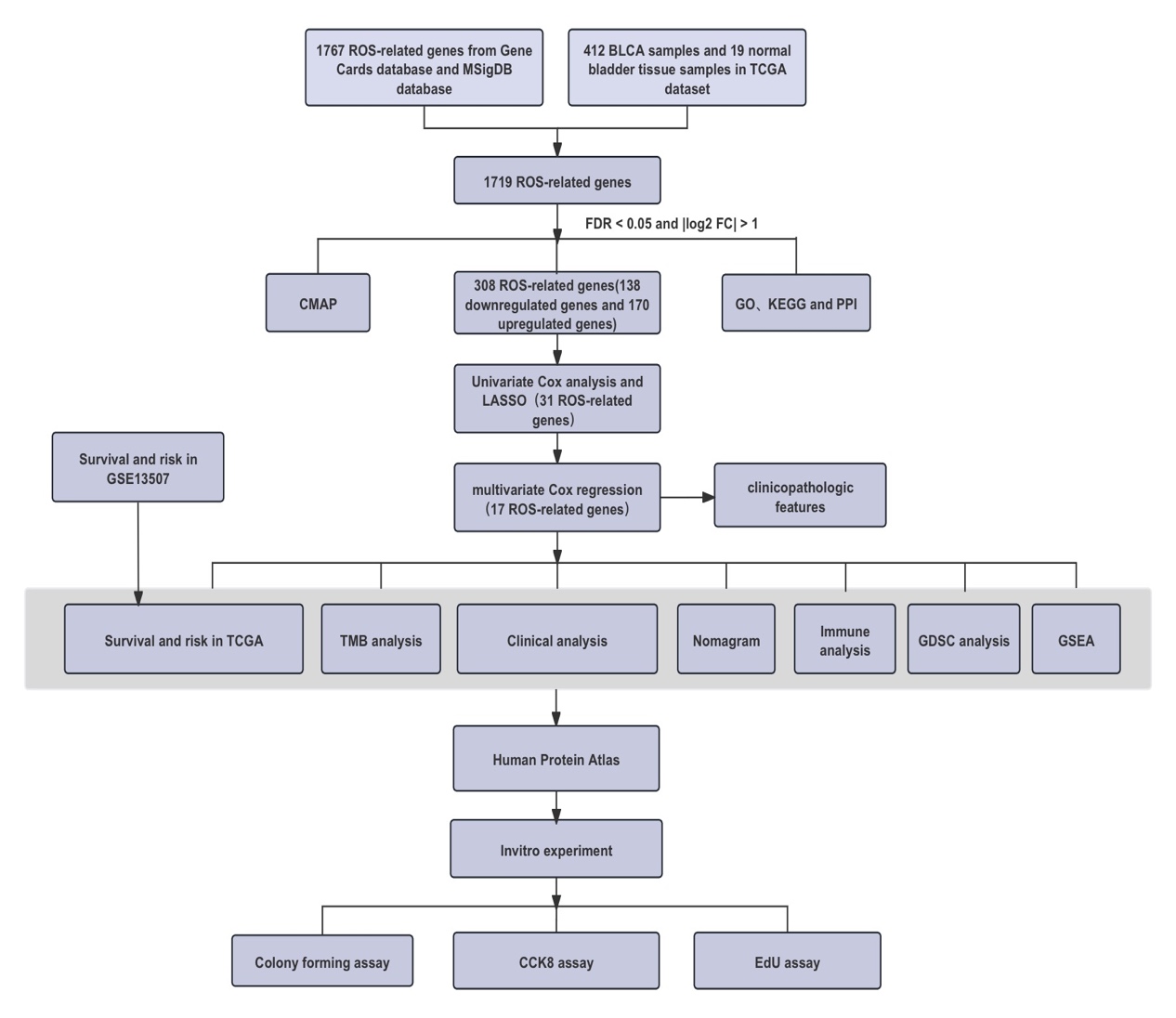
**Supplementary Figure 1.** The chief flow chart.


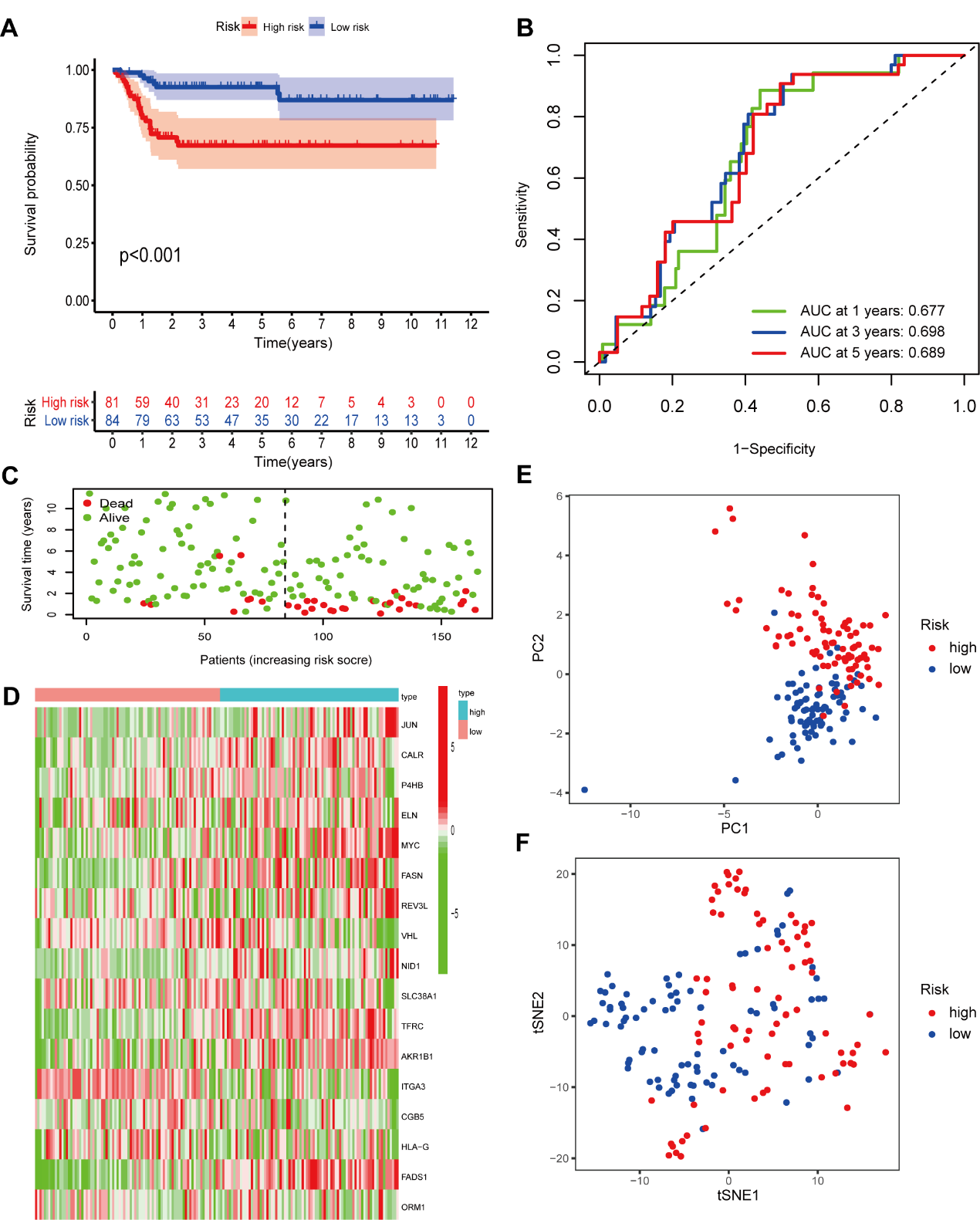


**Supplementary Figure 2.** Validation of prognostic ROS-based signature in GSE13507 dataset. (A) Kaplan-Meier survival analysis of BLCA patients between different groups; (B) Distribution of survival status based on the median risk score; (C)Time-independent receiver operating characteristic (ROC) analysis of risk scores for the 5-year survival; (D) Heatmap showed the differences of 17 ROS-related genes between different groups. (E) PCA analysis; (F)t-SNE analysis.
